# Learning Parsimonious Classification Rules from Gene Expression Data Using Bayesian Networks with Local Structure

**DOI:** 10.3390/data2010005

**Published:** 2017-01-18

**Authors:** Jonathan Lyle Lustgarten, Jeya Balaji Balasubramanian, Shyam Visweswaran, Vanathi Gopalakrishnan

**Affiliations:** 1 Red Bank Veterinary Hospital / 2051 Briggs Rd, Mt Laurel, NJ 08054, USA; 2 Intelligent Systems Program, University of Pittsburgh / 5113 Sennott Square, 210 South Bouquet Street, Pittsburgh, PA 15260, USA; 3 Department of Biomedical Informatics, University of Pittsburgh / 5607 Baum Boulevard, Pittsburgh, PA 15206, USA

**Keywords:** rule based models, gene expression data, bayesian networks, parsimony

## Abstract

The comprehensibility of good predictive models learned from high-dimensional gene expression data is attractive because it can lead to biomarker discovery. Several good classifiers provide comparable predictive performance but differ in their abilities to summarize the observed data. We extend a Bayesian Rule Learning (BRL-GSS) algorithm, previously shown to be a significantly better predictor than other classical approaches in this domain. It searches a space of Bayesian networks using a decision tree representation of its parameters with global constraints, and infers a set of IF-THEN rules. The number of parameters and therefore the number of rules are combinatorial to the number of predictor variables in the model. We relax these global constraints to a more generalizable local structure (BRL-LSS). BRL-LSS entails more parsimonious set of rules because it does not have to generate all combinatorial rules. The search space of local structures is much richer than the space of global structures. We design the BRL-LSS with the same worst-case time-complexity as BRL-GSS while exploring a richer and more complex model space. We measure predictive performance using Area Under the ROC curve (AUC) and Accuracy. We measure model parsimony performance by noting the average number of rules and variables needed to describe the observed data. We evaluate the predictive and parsimony performance of BRL-GSS, BRL-LSS and the state-of-the-art C4.5 decision tree algorithm, across 10-fold cross-validation using ten microarray gene-expression diagnostic datasets. In these experiments, we observe that BRL-LSS is similar to BRL-GSS in terms of predictive performance, while generating a much more parsimonious set of rules to explain the same observed data. BRL-LSS also needs fewer variables than C4.5 to explain the data with similar predictive performance. We also conduct a feasibility study to demonstrate the general applicability of our BRL methods on the newer RNA sequencing gene-expression data.

## 1. Introduction

Predictive modeling from gene expression data is an important biomedical research task that involves the search for discriminative biomarkers of disease states from a high-dimensional space. Comprehensible models are necessary in order to easily extract the predictive biomarkers from learned classifiers. The number of markers is in the order of several thousand measurements made from much smaller numbers of bio-specimens, often leading to several models that are equally good at the predictive task but differ in their abilities to summarize the observed data.

We have previously demonstrated that rule learning methods can be successfully applied to biomarker discovery from such sparse biomedical data [1–7]. Recently, we developed and extensively evaluated a novel probabilistic method for learning rules called Bayesian Rule Learning (BRL) [7]. This BRL algorithm was shown to perform on par or better than three state-of-the-art rule classifiers (Conjunctive Rule Learner[8], RIPPER[9], C4.5[10]) using 24 biomedical datasets. Therein, BRL was shown to outperform even C4.5, which was the best among the other methods. BRL used a global search of the space of possible constrained Bayesian network structures to infer a set of classification rules containing a posterior probability representing their validity. These rules are readily comprehensible and contain biomarkers and their cut-off values that discriminate the class variable.

In this paper, we relax some of the constraints from the BRL search space of global structures by introducing the more generalized local structure search that we call BRL-LSS (Bayesian Rule Learning- Local Structure Search). Henceforth, we refer to the global structure search as BRL-GSS (Bayesian Rule Learning- Global Structure Search) to distinguish it from BRL-LSS. We hypothesize that the more general local structure would lead to more parsimonious rule sets that enhance the comprehensibility of the rule model while maintaining the classification performance.

In this work, we develop an algorithm to perform this local structure search, the BRL-LSS, and evaluate it for parsimony and classification performance using more recently extracted gene expression data from public repositories. We hypothesize that the more generalized representation obtained from the local structure search results in a more parsimonious set of rules that describes the observed data as well as the global structure. Parsimony in the rule set representation contributes towards model comprehensibility by presenting a more concise summary of the observed data. In the Materials and Methods section of this paper, we introduce the BRL algorithm followed by a description of the global and local structure search. We then describe our experimental design to test our hypothesis and observe the results in the subsequent sections.

## 2. Results and Discussion

Table 1 shows the summary of the predictive performance of the tested classifiers averaged across the 10 folds of the cross-validation study. For each of the 10 datasets, we report the AUC and the Accuracy. We average the results across the 10 datasets in the bottom of the table. We also provide the standard error of the mean.

In terms of predictive performance each of BRL-GSS, BRL-LSS, and C4.5 appear to be comparable. There seems to be a fractional gain in performance by BRL-LSS with an average AUC of 0.775.

Table 2 shows the summary of the parsimony statistics of the tested classifiers averaged across the 10 folds of the cross-validation study. For each of the 10 datasets, we report the Number of rules in the rule base and the number of variables used. We provide the average and standard error of mean at the bottom of the table.

The results show that BRL-LSS makes a notably more parsimonious than BRL-GSS. It has fewer average number of rules, 5.76, when compared to BRL-GSS, which needs an average of 53.61 rules to obtain similar performance. The number of rules in BRL-LSS is almost comparable to the state-of-the-art classifier, C4.5, with an average of 4.77 rules. BRL-LSS needs fractionally more number of variables to meet the predictive performance of BRL-GSS. It uses an average of 4.37 variables, while BRL-GSS needs fractionally fewer variables at an average of 3.84. However, C4.5 needed almost twice as many variables (average of 8.49) to obtain the same performance as BRL-LSS.

To summarize, in terms of classification tasks where model parsimony is important (eg. Biomarker discovery) BRL-LSS can be preferred since it selects fewer variables than C4.5, needs fewer rules than BRL-GSS, while having very similar predictive power as the two.

### 2.1. Case Study

In this sub-section, we analyze the RNA-Seq dataset (KIRC) as described in section 2.2.2. We run BRL-GSS, BRL-LSS, and C4.5 on the KIRC dataset to learn predictive models over 10-fold cross-validation. We observe that the task of differentiating the tumor gene expression from matched normal samples is an easy task for the three algorithms. Each of the tested classifiers were evaluated using the predictive performance metrics (AUC and Accuracy) and the model parsimony metrics (average number of rules and variables used). BRL-LSS emerged as the best predictor with AUC = 0.984 (Accuracy = 99.17%), BRL-GSS as the next best with AUC = 0.975 (Accuracy = 98.69%), and C4.5 achieved an AUC = 0.961 (Accuracy = 98.35%).

We evaluate model parsimony by the average number of rules and variables appearing in models across 10-fold cross-validation. BRL-GSS on average required 10 rules and 2.4 variables. BRL-LSS required fewer rules on average: 7.7 rules with more variables (2.9). C4.5 required the least number of rules (4.3 on average), but needed the largest number of variables on average (7.2 variables) to model the data.

With high performing models, there can be several models that can perform more or less equally well. So, there can be different rule sets learned with BRL (and C4.5) composed of other variables that can match the performance achieved by the greedy best-first search algorithm. We now observe the results we obtained from the greedy best-first algorithms: BRL-GSS and BRL-LSS. The models were learned on the entire KIRC training dataset.

**Figure 1:**
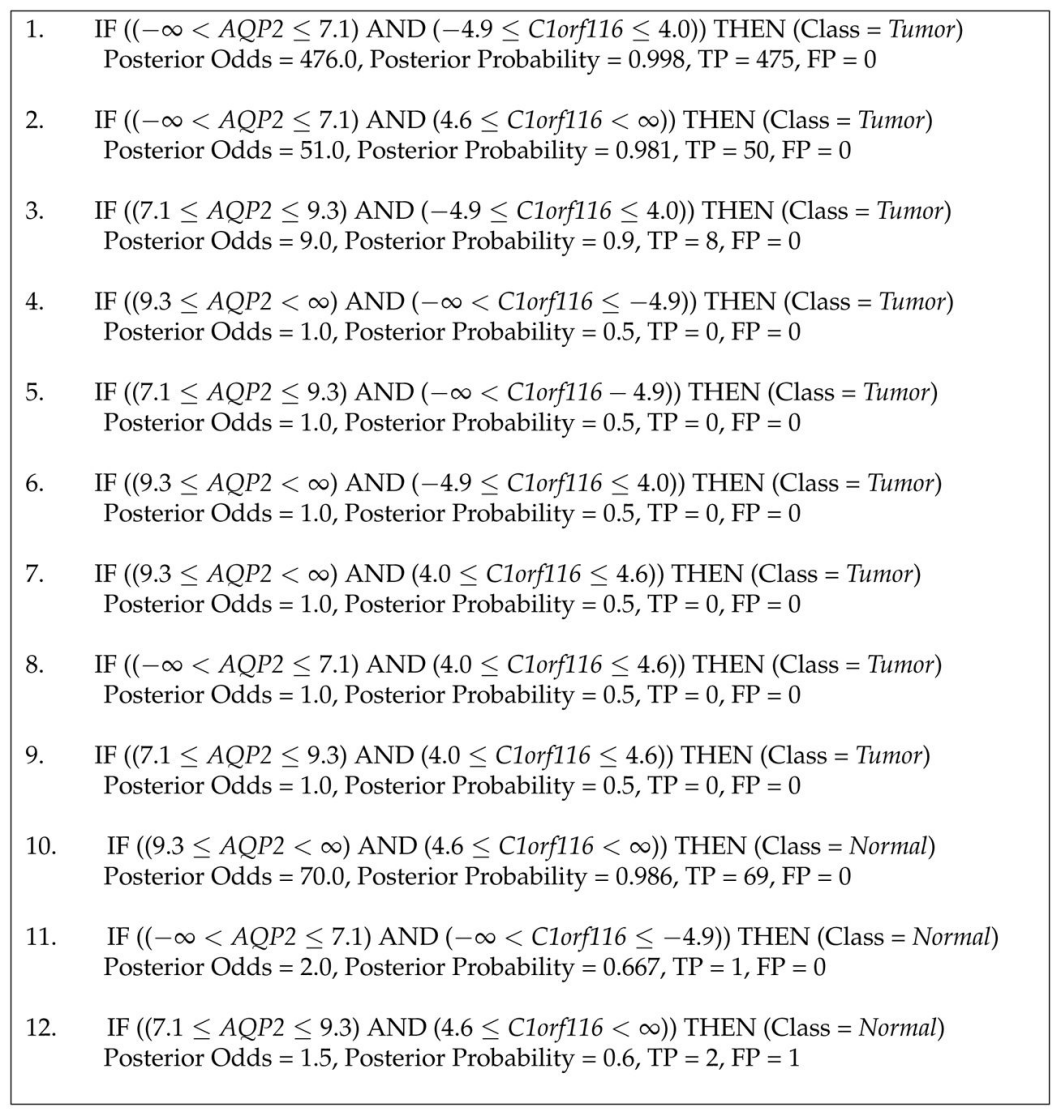
Rule set learned by BRL-GSS on the entire KIRC training data.

The rule set learned by BRL-GSS is shown in Figure 1. It uses two variables (genes): *APQ2* and *C1orf116. APQ2* takes three discrete values (as determined by EBD during discretization). *C1orf116* takes four discrete values. As expected, this generates twelve rules (four times three). Each rule also shows the number of true positives (TP) and false positives (FP) as computed by the rule on the training dataset. The posterior probability is computed by the smoothed expression– $\frac{\left(TP+1\right)}{\left((TP+1)+(FP+1)\right)}$. The posterior odds are the odds of the rule assigning the predicted class against all other classes– $\frac{\left(TP+1\right)}{\left(FP+1\right)}$. We notice that rules 3 through 9 have no evidence assigned from the training dataset. BRL produces rule models that are mutually exclusive and exhaustive. This means that for a given test instance, BRL explains the instance using utmost and at least one rule. The consequence is having rules with no evidence in the training dataset. This was the primary motivation for the development of BRL-LSS that limits the creation of these branches by merging them.

**Figure 2:**
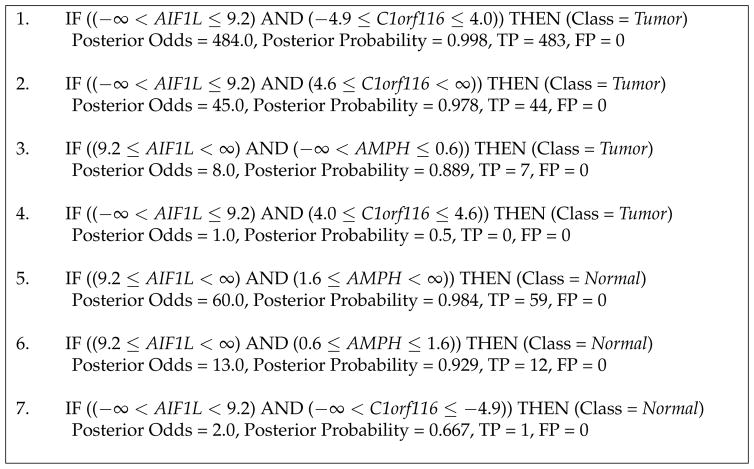
Rule set learned by BRL-LSS on the entire KIRC training data.

We observe this change in the rule set learned by BRL-LSS as shown in Figure 2. We immediately notice that the number of rules required by the model is fewer but needs more variables to explain the data. In this scenario, it also leads to an improvement in performance. The BRL-LSS model uses three variables (genes): *AIF1L*, *AMPH* and *C1orf116. AIF1L* takes two discrete values (as determined by EBD during discretization). *AMPH* takes three discrete values. *C1orf116* takes four discrete values. Note that a BRL-GSS model with the same performance as this BRL-LSS model would require 24 rules(two times three times four) that is largely composed of rules with no evidence in the training dataset. Using BRL-LSS, we manage to maintain the property of the rule set being mutually exclusive and exhaustive while achieving parsimony. The BRL-LSS rule set only requires 7 rules to describe the training data, while needing 3 variables to do so. We still end up with rules with no evidence (rule 4) but they are much fewer.

The purpose of this case study was to demonstrate the application of BRL-GSS and BRL-LSS in data from RNA-Seq technology. A complete data analysis of the KIRC dataset would involve further exploratory data analysis and examination of multiple rule sets to explain different hypothesis. Such thorough analysis of this dataset is beyond the scope of this paper.

## 3. Materials and Methods

### 3.1. Bayesian Rule Learning

A classifier is learned from gene expression data to explain disease states from historical data. The variable of interest that is predicted is called the *target variable* (or simply the *target*), and the variables used for prediction are called the *predictor variables* (or simply *features*).

Rule-based classifiers are a class of easily comprehensible supervised machine learning models that explain the distribution of the target, in the observed data, using a set of IF-THEN rules described using predictor variables. The ’IF’ part of the rule specifies a condition, also known as the *rule antecedent*, which if met, fires the ’THEN’ part of the rule, known as the *rule consequent*. The rule consequent makes a decision on the class label, given the value assignments of the predictor variables met by the rule antecedent. A set of rules is called a *rule base*, which is a type of knowledge base. The C4.5 algorithm learns a decision tree, where each path in the decision tree (from root of the tree to each leaf) can be interpreted as a rule. Here the variables selected in the path compose the rule antecedents as a conjunctions of predictive variable and value assignment to those variables. We infer a rule consequent based on the distribution of instances over the target that match this rule antecedent.

Bayesian Rule Learning (BRL) infers a rule base from a learned Bayesian network (BN). BN is a probabilistic graphical model with two components— a graphical structure, and a set of probability parameters [11]. The graphical structure consists of a directed acyclic graph. Here, the nodes represent variables and variables are related to each other by directed arcs that do not form any directed cycles. When there is a directed arc from node *A* to node *B*, node *B* is said to be the *child node*, and node *A* is said to be the *parent node*. A probability distribution is associated with each node, *X*, in the graphical structure given the state of its parent nodes, *P*(*X*|*Pa*(*X*)), where *Pa*(*X*) represents the different discrete value assignments of the parents of node *X*. This probability distribution is generally called a conditional probability distribution (*CPD*). For discrete-valued random variables, the CPD can be represented in form of a table called conditional probability table (*CPT*). Furthermore, any CPT can be represented as a rule base. Here, we consider only the CPT for the target variable. Each possible value assignments of the parents represent a different rule in the rule base. The evidence in form of the distribution of instances, for each target value, in the training data helps infer the rule consequent. The resulting rule base consists of rules that are mutually exclusive and exhaustive. In other words, at least one rule from the rule base matches a given instance and only one rule matches that instance.

We learn a BN from a training dataset using a heuristic search of the decision tree that results from the CPT described above. We evaluate how likely our learned BN generated the observed data using the Bayesian score (the K2 metric [12]). We demonstrated this process in our previous work [7].

Decision trees are popular compact representations of the CPT of a node in a BN. Most of BN literature is dedicated to learning global independence constraints in the domain. The global constraints only capture the dependent and independent variables that are parents to the node in the graphical representation. The number of parameters needed to describe the CPT is the number of joint assignments for the different parent variables of the node. The size of this CPT grows combinatorially to the number of parents of the node. As an example, let us consider a node representing a disease state. Let there be 10 genes (henceforth when we mention gene as a variable we are referring to its expression) that lead to the change of disease state. Let each gene take two discrete values (UP: up-regulated, DOWN: down-regulated). This would require 2^10^ = 1024 parameters to be represented by the CPT. Consequently our rule base has 1024 rules, one for each value assignment of the parent variables. Biomedical research, especially gene expression data rarely have enough training data to provide sufficient evidence to make class inference from the 1024 rules in our example scenario. It is therefore important to come up with a more efficient representation of the CPT.

#### 3.1.1. Bayesian Rule Learning- Global Structure Search (BRL-GSS)

We constrained our model to only those models with variables being a direct parent of the target variable. BRL uses breadth-first marker propagation (BFMP) for this algorithm, which provides significant speed up since database look-up is an expensive operation [13]. BFMP [13] permits bi-directional look-up using vectors of pointers by linking a sample to its respective variable-values, and the variable value to those samples that have it. It enables efficient generation of counts of matches for all possible specializations of a rule using these pointers.

**Figure 3:**
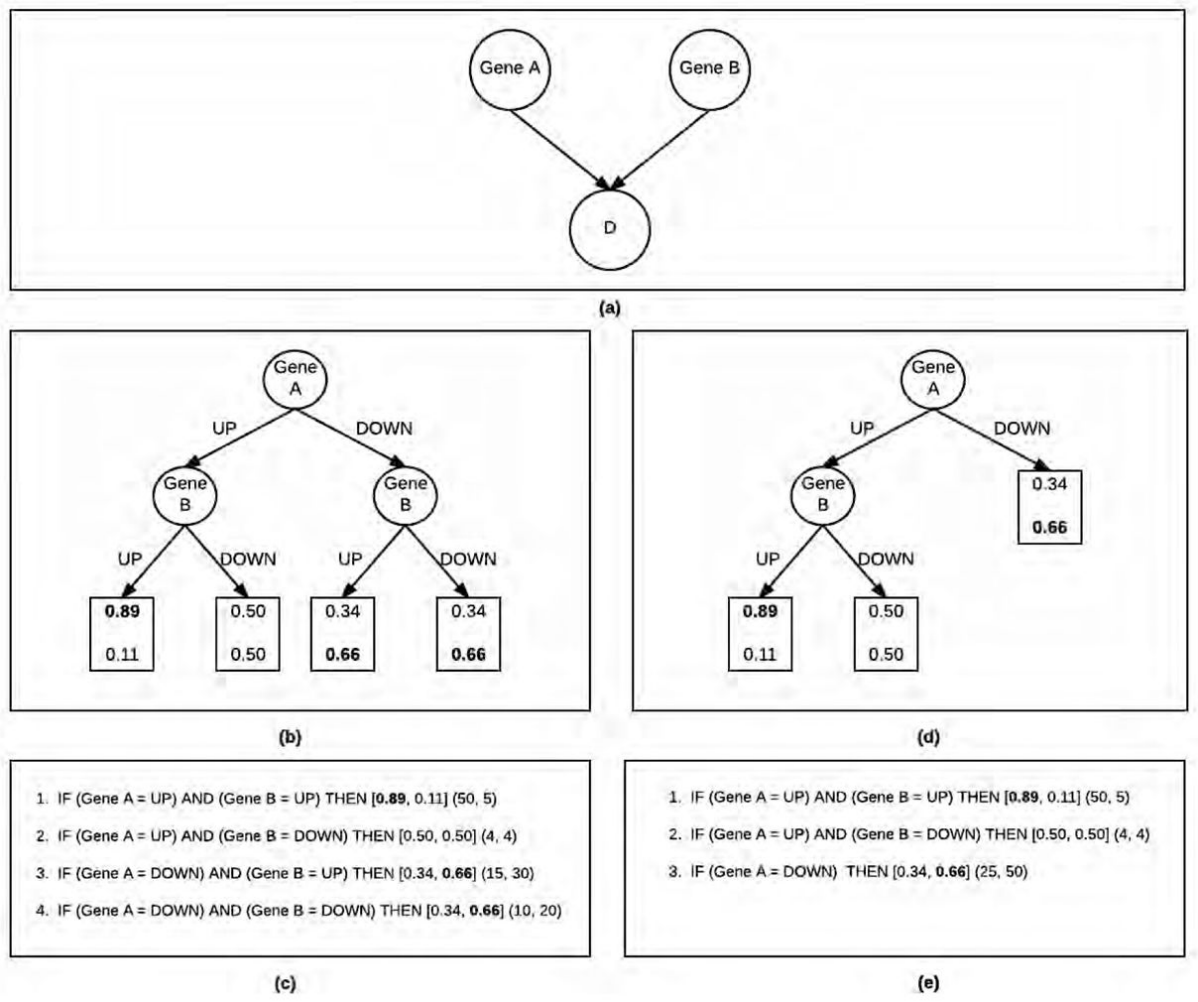
Bayesian Rule Learning (a) Bayesian Network for target *D* and predictor variables Gene *A* and gene *B*. (b) **BRL-GSS** CPT represented as a decision tree with *global* constraints. (c) **BRL-GSS** Decision tree represented as a BRL rule base. (d) **BRL-LSS** CPT represented as a decision tree with *local* constraints. (e) **BRL-LSS** Decision tree represented as a BRL rule base.

Figure 3 depicts a BN (3a), the corresponding global CPT representation using decision tree (3b), and the corresponding rules to the decision tree (3c). The BN in Figure 3a is a Bayesian network with one child variable also the target, *D*, and two parent variables (the predictor variables), Gene *A* and Gene *B*. Each predictor variable, Gene *A* and Gene *B* is binary. When the gene is up regulated they take the value *UP*. When the gene is down-regulated, they take the value *DOWN*. Figure 3b represents the CPT represented as a decision tree with global constraints. Since, both the predictor variables are binary, the decision tree has 2^2^ = 4 parameters, each represented by a leaf in the decision tree. Each leaf of the decision tree is a parameter, the conditional probability distribution over the target, given the values assigned by the path in the tree. This distribution for target *D* is shown in the leaf node. For example, given that Gene *A* takes value *UP* and Gene *B* takes value *UP*, the probability of *D* = *true* is 0.89, while probability of *D* = *f alse* is 0.11.

Figure 3c depicts a decision tree represented as a rule base. The rule antecedent (IF part) contains a conjunction of predictor variable assignments as shown in the path of the decision tree. The rule consequent is the conditional probability distribution over the target values (in square brackets) followed by the the distribution of the instances from the training data for each target value that match the rule antecedent. In rule 1, we see the evidence to be (50, 5) where there are 50 instances in the training dataset that matches the rule antecedent that have value *D* = *true*. There are only 5 instances that matches the rule antecedent that have value *D* = *f alse*. They are then smoothed with a factor *α*, set to 1 as a default. This simplifies the posterior odds to the ratio of (*TP* + *α*)/(*FP* + *α*), where TP is the number of true positives for the rule (where both the antecedent and consequent match with the test instance) and FP is the number of false positives (antecedent matches, but consequent does not match the test instance).

During prediction the class is determined by simply the higher conditional probability. In our example, since *D* = *true* has a probability of 0.89, the prediction for a test case that matches this rule antecedent is *D* = *true*. If there is a tie, by default the value of the majority class is the prediction.

We developed and tested two variants of global structure search using BRL, the *BRL*_1_ and *BRL*_1000_ in [7]. The subscripts indicate the number of BN models that are kept in memory during the best-first search. We concluded that *BRL*_1000_ was statistically significantly better than *BRL*_1_ and C4.5 on Balanced Accuracy, and RCI (relative classifier information). For this paper, we choose the *BRL*_1000_ version of the algorithm and rename it to BRL-GSS to be consistent with nomenclature for the local structure search algorithm we present in the next section. The worst-case time-complexity of BRL-GSS, for a dataset with *n* variables and *m* instances, where each variable *i* has *r_i_* discrete values and *r* = max *r_i_*, is *O*(*n*^2^*mr*). If we constrain the maximum number of discrete values that a variable can take on (for example, assume all variables are binary-valued), then the time-complexity reduces to *O*(*n*^2^*m*).

#### 3.1.2. Bayesian Rule Learning- Local Structure Search (BRL-LSS)

We adapted the method developed by [14] which can be used for developing an entire global network based on local structure. In Figure 3a, we see the same BN with two parents as the one we saw in BRL-GSS. Figure 3d, shows the local decision tree structure. In Figure 3, we saw that the distribution of the target, when Gene *A* = *DOWN* is the same regardless of the value of Gene *B*. To be precise, *P*(*D*|*GeneA* = *DOWN*, *GeneB* = *UP*) = *P*(*D*|*GeneA* = *DOWN*, *GeneB* = *DOWN*) = [0.34, 0.66]. The more general representation in Figure 3d, merges the two redundant leaves to provide a single leaf. As a result Figure 3e, reduces the number of rules to 3 down from 4. So, Figure 3e is said to be a more parsimonious representation of the data when compared to Figure 3c.

Next, we describe our algorithmic implementation to learn local decision trees as seen in Figure 3d. At a high level, our algorithm initializes a model with a single variable (gene) node as the root. For each unique variable in the dataset, there can be a unique root at the decision tree. A leaf in the initial model represents a specific value assignment of the root variable. By observing the classes of instances in the dataset that match this variable value assignment, we infer the likely class of an instance that would match this variable value assignment. To evaluate the overall model, we use the Bayesian Score to evaluate the likelihood that this model generated the observed data. The algorithm then iteratively explores further specialized models by adding other variables as nodes to one of the leaves of the decision tree. The model is then re-evaluated using the Bayesian Score. The model space here is huge at *O*(*n*!). Our algorithm adds some greedy constraints to bring the space down. In the following paragraphs, we specify how we constrain the search.

Algorithm 1 is the pseudocode of the local structure search module in the BRL. This algorithm takes as input, the data *D* and two parameters *maxConj* and *beamWidth* similar to BRL-GSS. We also used the heuristic of maximum number of parents (*maxConj*) to prevent overspecialization as well as to reduce the running time (default is set to 8 variables per path). The *beamWidth* parameter is the size of the priority queue (*beam*) that limits the number of BNs that the search algorithm stores in memory at a given step of the search. This beam sorts the BNs in reducing order of their Bayesian score. Line 2 initializes this beam with *singleton models*. These BNs have a single child and a single parent. The child is fixed to be the target. The parent is set iteratively to all the predictor variables in the training data *D*. During the search, this initial parent variable is set as the root node of the tree. A variable node is split in two ways— 1) Binary split and 2) Complete split. In binary split the variable is split into two values. If the variable has more than two discrete values (say |*v*|), the binary split creates $\left(\begin{array}{c} |v|\\ 2 \end{array}\right)$ different combinations of local decision trees. The complete split generates |*v*| different paths, one for each discrete value of the variable.

In line 3, the search algorithm specializes each model on the beam by adding a new parent variable as a candidate conjunct for each leaf in the decision tree. The best models from this specialization step are added to the final beam (line 6), which keeps track of the best models seen by the search algorithm so far. Line 7 checks to ensure that any candidate models for further specialization do not exceed the *maxConj* limit for the number of parents of the target in the BN. The loop at line 8 iterates through each unexplored variable in *D* for specialization. The loop in line 10 iterates through all the leaves of the local structure decision tree inferred from the BN. From lines 11 through 17, the algorithm performs a binary and complete split using the variable currently being explored at the specific leaf of the decision tree. It stores only the best model (as determined by the Bayesian Score) seen in this iteration.

Lines 18 through 21 check if the specialization process led to an improvement (better Bayesian score) to the model it started with. If the score improves, the new model is queued for further specialization in the subsequent iterations of the search algorithm.

Finally, in line 23 the best model seen during the search so far is returned by the search algorithm. This best-first search algorithm uses a beam to search through a space of local structured CPTs of BNs. As described in Figure 3e, BRL interprets this decision tree as a rule base.

**Algorithm 1:** Bayesian Local Structure Search

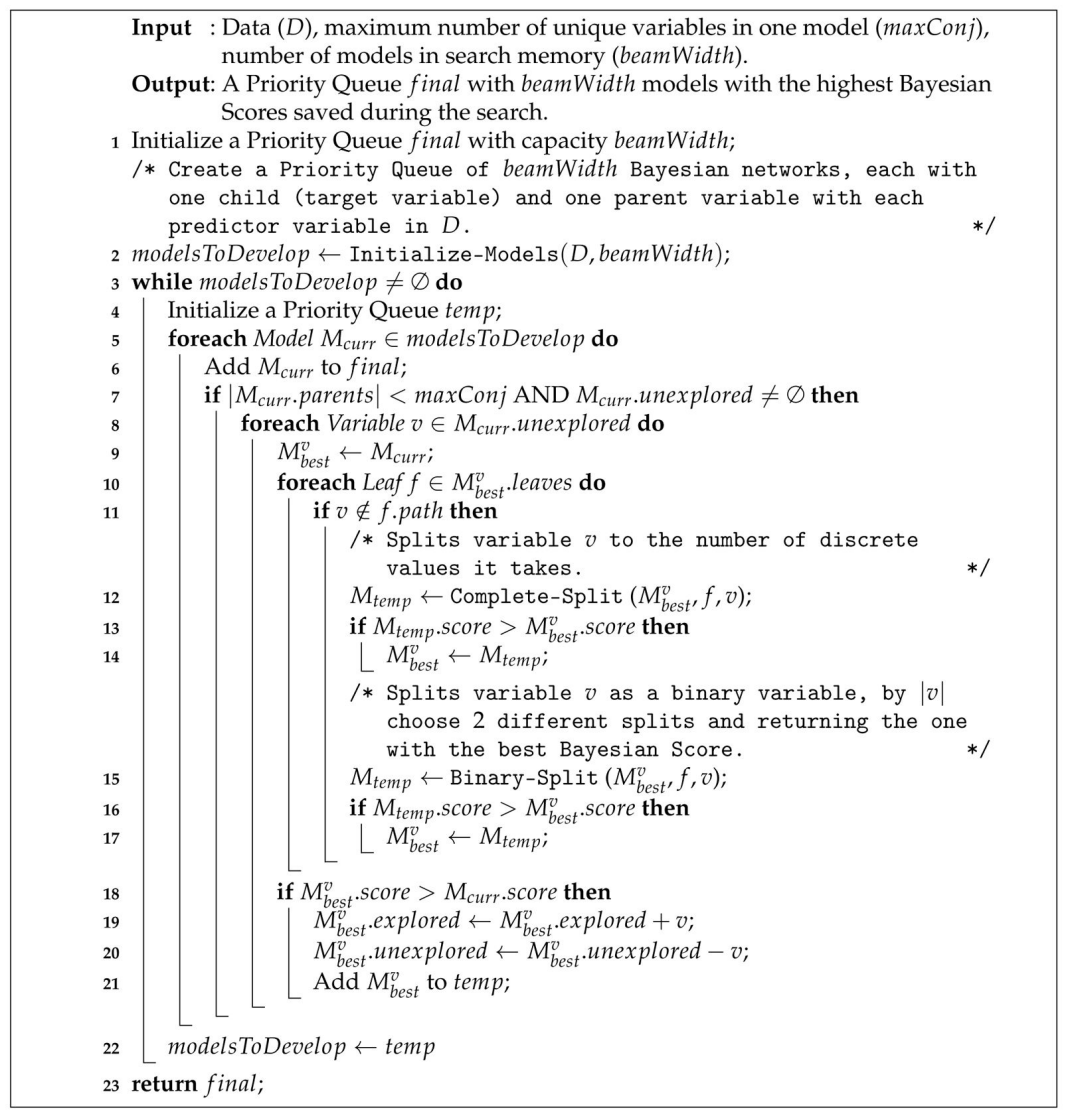

The worst-case time-complexity of BRL-LSS remains *O*(*n*^2^*mr*) as with BRL-GSS.We achieve this by the same global constraint on the maximum number of parents that the model can have in line 7 of the algorithm. However, in practice BRL-LSS tends to be generally slower than BRL-GSS. This is because in BRL-GSS we keep track of the variables already explored, for the entire beam. In BRL-LSS we keep track of the explored variables for each model separately. We still only have a constant number of models as constrained by the beam width. As a result the worst-case time-complexity remains the same as BRL-GSS. If we restrict the maximum number of discrete values each variable can take, the complexity reduces to *O*(*n*^2^*m*). As a result, with BRL-LSS we now explore a much richer space of models with the same time-complexity as BRL-GSS.

### 3.2. Experimental Design

For each biomedical dataset, we split the data into train and test set using cross-validation split. BRL-GSS and BRL-LSS require discrete data so, the training dataset is discretized. After learning the discretization scheme for each of the features from the training data, we apply the discretization scheme on those features in the test dataset. Finally, we learn a rule model from our different algorithms on the training data. We use this model to predict on the test data and we evaluate our performance. The detailed description on the cross-validation design, discretization method, classification algorithms, and performance metrics used for evaluation is described below.

#### 3.2.1. Classification Algorithms

We test three algorithms in the modeling step of the experimental design framework, to generate our rule models— 1) The *BRL-GSS*, which was the significantly best model from our previous study [7] comparing other state-of-the-art rule models; 2) *BRL-LSS*, which is our proposed method in this paper with a promise on model parsimony; and finally 3) We have shown previously [7] that C4.5 outperforms other readily available rule learners, and therefore for the purposes of comparison in this paper, we consider C4.5 as state-of-the-art. Decision trees can be translated into a rule base by inferring a rule from each path in the decision tree. C4.5[10] is the most popular decision tree based method. It was an extension to an earlier ID3 algorithm.

Both the BRL methods take in two parameters— *maxConj*, maximum number of features used in the Bayesian Network model, and *beamWidth*, maximum number of models stored in the search memory. For both BRL-GSS and BRL-LSS, we set *maxConj* = 8, and *beamWidth* = 1000. These were arbitrary choices that we use as defaults for the BRL models. The C4.5 also uses its default parameters as set by Weka.

#### 3.2.2. Dataset

We run our experiments on 10 binary class, high-throughput, biomedical data. Each of the 10 datasets chosen here represent a cancer diagnostic problem of distinguishing cancer patients from normal patients using their gene expression profile. The gene expression data is generated from high-throughput microarray technology. Table 3 shows the dataset dimensions and sources for the 10 datasets.

In addition to the high-throughput microarray technology data for gene expression used in our experiments, we also conduct a case study using data generated from the newer RNA-sequencing (RNA-Seq) technology for gene expression. We obtain Illumina HiSeq 2000, RNA-Seq Version 2, normalized, gene expression data of patients with Kidney Renal Clear Cell Carcinoma (KIRC), processed using the RNA-Seq Expectation Maximization (RSEM) pipeline from The Cancer Genome Atlas (TCGA)[23]. The samples are primary nephrectomy specimens obtained from patients with histologically confirmed clear cell renal cell carcinoma and the specimens conform to the requirements for genomic study by TCGA. We develop a model to differentiate the gene expression in tumor samples from matched normal samples (normal samples from patient with the tumor). This KIRC dataset has 606 samples (534 tumor, 72 normal) and 20531 mapped genes.

We pre-process the KIRC dataset by removing genes with sparse expression (more than 50% of the samples have value 0). We are left with 17694 genes. As recommended in RNA-Seq analysis literature [24], we use Limma’s voom transformation[25] to remove heteroscedasticity from RNA-Seq count data and to be unaffected by outliers in the data. In this case study described in the results section 3.1, we demonstrate the feasibility of our rule learning methods in analyzing RNA-Seq data.

As described in the experimental design framework, our datasets need to be discretized for applying our algorithms. All the biomedical datasets in Table 3 contain continuous measurements of the markers. Each training fold of data is discretized using the efficient Bayesian discretization method (EBD) [26] with a default parameter, *λ* = 0.5, which controls the expected number of cut-points for each variable in the dataset.

#### 3.2.3. Evaluation

For each of the 10 datasets, we performed a 10-fold stratified cross-validation for sampling from a dataset. We measure each performance metric (described below) for each fold in the cross-validation and then average that metric across the 10 folds to get an estimate of that performance metric.

We measure 4 performance metrics. We use 2 metrics to evaluate the classifier predictive performance, and another 2 to evaluate model parsimony. The first metric for classifier predictive performance is the *Area Under the Receiver Operator Characteristics Curve* (AUC). It indicates the class discrimination ability of the algorithm for each dataset. It ranges from 0.0 to 1.0. Higher value indicates a better predictive classifier. The second metric for classifier performance is *Accuracy*, given as a percentage. Again higher value indicates a better predictor.

The first parsimony metric is the *Number of Rules*. All the algorithms tested have rule bases that are mutually exclusive and exhaustive. This means that each instance in the dataset is covered by at least one rule, and exactly one rule. A small number of rules in the rule base indicates greater coverage by individual rules. The coverage of a rule is the fraction of the instances in the training data that satisfies the antecedent of the rule. A large number of rules indicates that each rule have small coverage, and as a result lesser evidence. A small number here is attractive because a parsimonious model with few rules to describe all the observed data indicates generalized rules with stronger evidence per rule. The second parsimony metric is the *Number of Variables*. Typically in a biomarker discover task that involves a high-dimensional gene expression data, we would like fewer variables to describe the observed data. This is because the validation of those markers is time consuming and expensive. Having fewer variables to verify is appealing in this domain. So, we prefer fewer number of variables that gives us the best predictor.

## 4. Future Work

We previously extended BRL-GSS with a Bayesian Selective Model Averaged version called SMA-BRL[27]. We showed that SMA-BRL was a significantly better predictor than BRL-GSS. In the future, we would like to study the selective model averaged version of BRL-LSS. For the remainder of this paper, we refer to the collection of classifiers— BRL-GSS, BRL-LSS, and their Selective Model Averaged versions as the *BRL system*. The descriptive capability and predictive power enables us to envision the applications of the BRL system to other high-profile genomic problems. We discuss two possible applications in the following paragraphs.

An important problem in genomics is the classification of a SNP as either neutral or deleterious. Deleterious SNPs can disrupt functional sites in a protein and can cause several disorders. SNPdryad[28] is one such classifier that uses only orthologous protein sequences to derive features (Sequence Conservation Profile) that assist in this classification task. In addition to the Sequence Conservation Profile, they use other features like the physiochemical property of amino acids, the functional annotation of the region with the SNP, number of sequences in the multiple sequence alignment, and the number of distinct amino acids for the classification task. They compare 10 different classifiers for the task and report excellent predictive performance using Random Forests. However, they do not explore any classifier that offers readily interpretable descriptive statistics. We propose to explore this problem using the BRL system, since it readily offers descriptive statistics in form of rule sets. It would be interesting to learn which features lead to deleterious non-synonymous human SNPs.

Another problem of interest is the *in silico* evaluation of target sites for the CRISPR/Cas9 system. CCTop[29] is an experimentally validated tool that is used to select and evaluate targeting site for the CRISPR/Cas9 system. CCTop evaluates target sites in a genome by using a score derived from the likelihood of the stability of the heteroduplex (formed from the single guide RNA and the DNA) and the proximity of an exon to the target. BRL system can be used to learn rules that indicate a good target site for the CRISPR/Cas9 system. The classifiers in the BRL system are composed of rules, each with a posterior probability indicating the uncertainty in the validity of the rule. This probability score can be used to rank the target sites. In addition, it would be interesting to explore other candidate variables to improve the performance of the rule sets.

## 5. Conclusions

In this paper, we presented extensions to the BRL-GSS by relaxing the constraints on the decision tree representation using local structures of the conditional probability table of the learned Bayesian network. This led to the creation of BRL-LSS that explores a richer and more complex model space while maintaining the worst-case time-complexity with BRL-GSS. BRL-LSS is now incorporated as part of the BRL system, which is provided in the Supplementary materials. This system can be used for predictive modeling of any quantitative datasets, even though it has been developed primarily for the analysis of biomedical data. The advantages of this system over state-of-the-art machine learning classifiers include: 1) comprehensibility and ease in extracting discriminative variables/ biomarkers from interpretable rules, and 2) parsimonious models with comparable predictive performance, and 3) the ability to handle discretization of high-dimensional biomedical datasets using simple command line parameters integrated into the BRL system. We hope that BRL system will find applications in other challenging domains especially the ones with high-dimensional data.

## Figures and Tables

**Table 1:** Predictive performance using the Average and Standard Error of Mean (SEM) of Accuracy and AUC

Data ID	Average AUC			Average Accuracy		

	BRL-GSS	BRL-LSS	C4.5	BRL-GSS	BRL-LSS	C4.5
1	0.864	0.809	0.821	73.08	74.63	69.92
2	0.596	0.570	0.528	90.33	85.16	83.46
3	0.948	0.936	0.929	90.36	90.18	93.09
4	0.694	0.807	0.469	73.00	80.00	61.00
5	0.815	0.921	0.807	86.56	92.67	88.44
6	0.497	0.540	0.732	72.22	67.22	85.14
7	0.898	0.877	0.877	85.71	84.64	87.50
8	0.857	0.847	0.807	92.38	90.71	90.71
9	0.463	0.494	0.594	55.00	60.00	61.67
10	0.950	0.950	0.950	94.17	94.17	94.17

Average	0.758	0.775	0.751	81.28	81.94	81.51
SEM	0.06	0.05	0.05	3.96	3.62	3.98

**Table 2:** Model Parsimony using the Average and Standard Error of Mean (SEM) of the Number of Rules and Features

Data ID	Average Number of Rules			Average Number of Features		

	BRL-GSS	BRL-LSS	C4.5	BRL-GSS	BRL-LSS	C4.5
1	307.10	18.00	13.30	8.00	15.70	25.60
2	105.60	6.50	5.30	6.50	5.40	9.60
3	5.40	3.90	2.90	2.30	2.70	4.70
4	33.60	6.10	6.50	4.80	4.70	11.90
5	7.60	3.30	2.50	2.60	2.00	4.00
6	42.40	6.30	5.30	5.10	4.30	9.40
7	4.40	3.10	2.80	2.10	2.10	4.60
8	3.20	2.60	2.00	1.60	1.60	3.00
9	24.80	5.80	5.10	4.40	4.20	9.10
10	2.00	2.00	2.00	1.00	1.00	3.00

Average	53.61	5.76	4.77	3.84	4.37	8.49
SEM	29.89	1.46	1.08	0.72	1.34	2.15

**Table 3:** The 10 datasets used in our experiments (sorted by the number of instances). The columns indicate the data ID number, number of instances, number of features, the class label distribution among the instances, and the source of the data.

Data ID	# instances	# features	Class distribution	Source
1	249	12625	(201, 48)	[15]
2	175	6019	(159, 16)	[16]
3	103	6940	(62, 41)	[17]
4	100	6019	(76, 24)	[18]
5	96	5481	(75, 21)	Dr. Kaminski
6	86	5372	(69, 17)	[19]
7	72	7129	(47, 25)	[20]
8	63	5481	(52, 11)	Dr. Kaminski
9	60	7129	(40, 20)	[21]
10	36	7464	(18, 18)	[22]
